# Good-bye to the Doctor

**Published:** 1883-03-20

**Authors:** 


**Good-Bye to the Doctor.**—Bouvart, on entering one morning the chamber of a French marquis, whom he had attended through a very dangerous illness, was accosted by his noble patient in the following terms: "Good day, Mr. Bouvart; I feel quite in spirits, and think my fever has left me." I am sure it has," replied Bouvart, dryly. "The very first expression you used convinces me of it." "Pray explain yourself." "Nothing is easier. In the first days of your illness, when your life was in danger, I was your dearest friend; as you began to get better, I was your good Bouvart; and now I am Mr. Bouvart. Depend upon it, you are quite recovered."—*Louisville Medical News.*

